# Differential potentials of neural progenitors for the generation of neurons and non-neuronal cells in the developing amniote brain

**DOI:** 10.1038/s41598-019-40599-2

**Published:** 2019-03-14

**Authors:** Yuki Hashimoto, Hitoshi Gotoh, Katsuhiko Ono, Tadashi Nomura

**Affiliations:** Developmental Neurobiology, Graduate School of Medicine, Kyoto Prefectural University of Medicine, 1-5 Shimogamo-hangi cho, Sakyoku, Kyoto 606-0823 Japan

## Abstract

Mature mammalian brains consist of variety of neuronal and non-neuronal cell types, which are progressively generated from embryonic neural progenitors through the embryonic and postnatal periods. However, it remains unknown whether all embryonic progenitors equivalently contribute to multiple cell types, or individual neural progenitors have variable potentials to generate specific cell types in a stochastic manner. Here, we performed population-level tracing of mouse embryonic neural progenitors by using Tol2-mediated genome integration vectors. We identified that neural progenitors in early embryonic stages predominantly contribute to cortical or subcortical neurons than astrocytes, ependymal cells, and neuroblasts in the postnatal brain. Notably, neurons and astrocytes were cumulatively labeled by the increase of total labeled cells, suggesting constant neurogenic and gliogenic potentials of individual neural progenitors. On the contrary, numbers of labeled ependymal cell are more fluctuated, implicating intrinsic variability of progenitor potentials for ependymal cell generation. Differential progenitor potentials that contribute to neurons, astrocytes, and ependymal cells were also detected in the developing avian pallium. Our data suggest evolutionary conservations of coherent and variable potentials of neural progenitors that generate multiple cell types in the developing amniote brain.

## Introduction

**Figure 1:**
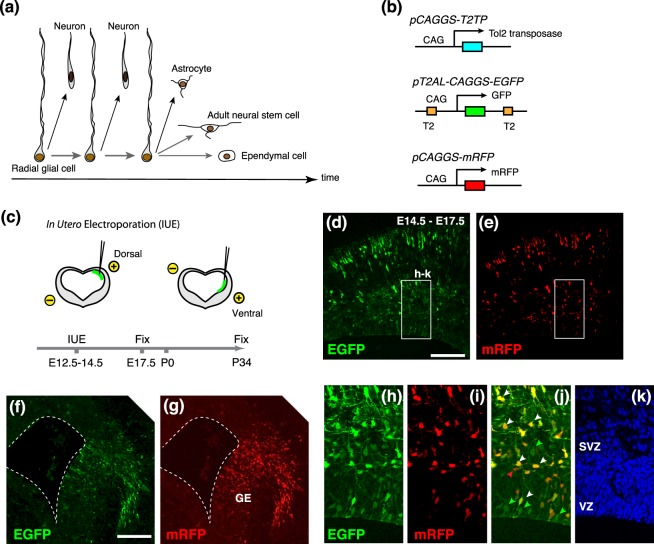
Population-level tracing of neural progenitors by using genome-integrative vectors. (**a**) Progressive changes in the potential of neural progenitor (radial glial cell) from embryonic to postnatal period. (**b**) Expression vectors for Tol2 transposase (pCAGGS-T2TP), EGFP flanked by Tol2-responsive elements (T2; pT2AL-CAGGS-EGFP), and mRFP (pCAGGS-mRFP), all of which are driven by CAG promoter. (**c**) Schematic drawings of *in utero* electroporation. Reporter vectors were introduced to the dorsal or ventral part of the embryonic mouse telencephalon at E12.5, E13.5 or E14.5. (**d**–**k**) Distributions of EGFP- and/or mRFP- positive cells in the neocortex (**d**,**e**,**h**–**k**) and the ganglionic eminence (GE; **f**,**g**) at 3 days after electroporation. In the ventricular and subventricular zones (VZ and SVZ), majority of labeled cells coexpressed EGFP and mRFP (white arrowheads), while a few cells were labeled by only EGFP (green arrowheads). A red arrowhead indicates mRFP single-positive cell (**j**). Scale bars: 200 µm.

Mature vertebrate brains comprise enormous number of neuronal and non-neuronal cells, from which complex neuronal circuits are assembled to produce higher-ordered behavioral and cognitive functions. All neurons and glial cells in brains are derived from embryonic and postnatal neural stem and progenitor cells^1,2^. In the developing mammalian telencephalon, neural progenitors (radial glial cells) residing in the ventricular zone (VZ) undergo self-renewal and concomitantly produce various types of projection or interneurons in spatially and temporally controlled manners. Subsequently, large numbers of glial cells, such as astrocytes and oligodendrocytes, are generated from neural progenitors during perinatal and postnatal periods. The remnants of ventricular neural progenitors differentiate into ependymal cells that line the postnatal ventricular wall. Furthermore, some of embryonic neural progenitors are maintained as postnatal/adult neural stem cells in the subventricular zone (SVZ) of the lateral ventricle, which contribute to persistent neurogenesis throughout animal life^3^ (Fig. 1a). The temporal sequences of neurogenic and gliogenic phases, as well as continuous neurogenesis in postnatal brains, are highly conserved in vertebrates, while numerous variations in neuron and glial cell types are evident among species^4,5^.

Several lines of evidence suggest that embryonic neural progenitors retain multi-potency to produce various types of neurons and glial cells; the range of progenitor potentials is thought to be progressively restricted to produce specific cell types in response to intrinsic and extrinsic factors^6–8^. In contrast, recent studies have demonstrated the heterogeneity of embryonic neural progenitors with respect to neurogenic and/or gliogenic potentials^9–13^. Furthermore, it has been shown that a slowly proliferating subpopulation of embryonic neural progenitors contributes to neural stem cells in the adult SVZ^14,15^. Almost equal numbers of neurons and glial cells exist in the adult mouse cerebral cortex^16^, suggesting that the neurogenic and gliogenic potentials of progenitors are tightly controlled during embryogenesis. However, it still remains unclear whether every embryonic neural progenitor retains an equivalent potential to generate multiple neuronal and non-neuronal cell types in the mature brain, or individual progenitors have variable potentials to generate specific cell types in a stochastic manner.

Here, we performed population-level tracing of mouse embryonic neural progenitors by Tol2 transposon-mediated genome integrating vector. We identified that neural progenitors in the early stages of the mouse telencephalon predominantly contribute to cortical or subcortical neurons rather than astrocytes, ependymal cells and neuroblasts in the rostral migratory stream (RMS). Notably, the number of labeled neurons and astrocytes was cumulatively increased according to the total number of labeled cells, suggesting that majority of progenitors has similar probabilities to generate neurons and astrocytes. In contrast, numbers of labeled ependymal cells were more fluctuated, implicating limited number of progenitors contributed to ependymal cells. Furthermore, similar trends of neurogenesis and gliogenesis were observed in the developing chick brain. Our data suggest that differential potentials of neural progenitors in the production of neurons and non-neuronal cells, and that common developmental mechanisms underlie the region- and time-dependent neurogenesis and gliogenesis in distantly related species.

## Results

### Population-level tracing of murine cortical neural progenitors with a genome-integration vector

To investigate lineage relationships of multiple cell types derived from embryonic neural progenitors, we utilized a transposon-derived vector (pT2AL-CAGGS-EGFP) that expresses enhanced green fluorescent protein under the control of a ubiquitous promoter (Fig. 1b)^17,18^. Introduction of this vector simultaneously with a Tol2 transposase-expression vector (pCAGGS-T2TP) into the developing brain provided permanent tracing of proliferative progenitors and their sibling cells^19–21^. In addition, co-electroporation of these vectors and a non-integrating reporter vector (pCAGGS-mRFP) enabled multiple cell types to be distinguished depending on their differentiation timing; it is expected that early-born cells are labeled with both EGFP and mRFP due to immediate expression of transfected vectors, while later-born cells are only labeled with EGFP due to genomic integration of the transposon-derived vector.

**Figure 2:**
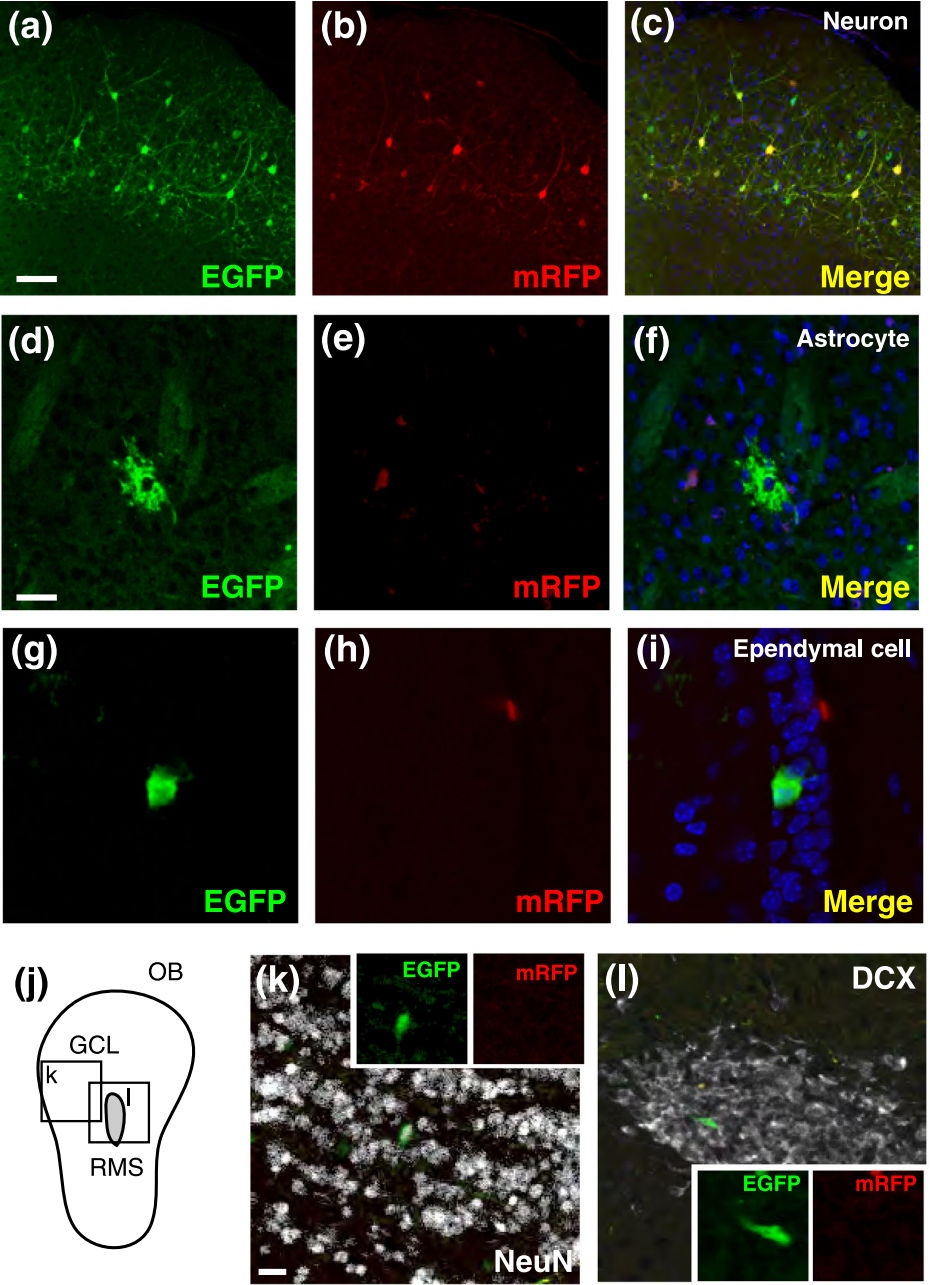
EGFP- and/or mRFP-positive cells in the P34 mouse brains. (**a**–**c**) Superficial layers of cortical neurons labeled with both EGFP and mRFP. (**d**–**i**) An astrocyte (**d**–**f**), and ependymal cell (**g**–**i**) labeled with EGFP but not mRFP. (**j**) Schematic drawing of the olfactory bulb (OB). (**k**,**l**) A NeuN-positive cell in the granular cell layer (GCL; **j**,**k**) and a DCX-positive neuroblast in the rostral migratory stream (RMS; **j**,**l**) that were labeled with EGFP but not mRFP. Scale bars: 100 µm in (**a**,**k**), 16 µm in (**d**).

**Figure 3:**
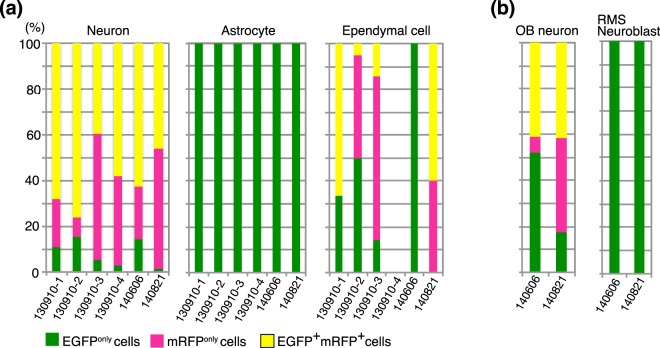
The proportions of EGFP- and/or mRFP-positive cells among multiple cell types in P34 mouse brains. (**a**,**b**) EGFP^only^, mRFP^only^, and EGFP^+^mRFP^+^ cells in neurons, astrocytes, ependymal cells, OB neurons, and RMS neuroblasts were quantified in P34 mouse brains. Astrocytes and RMS neuroblasts were exclusively labeled with EGFP. OB neurons and RMS astrocytes were detected only in two samples (140606 and 140821) in which the ventral telencephalon was targeted (**b**).

We electroporated these expression vectors into the E12.5, E13.5, and 14.5 mouse dorsal or ventral telencephalon (the pallium and subpallium, respectively) by changing the direction of the electrodes (Fig. 1c). To minimize the unbalanced expression of fluorescent reporters, equal amount of EGFP and mRFP vectors were introduced into the brain. At 3 days after electroporation, we confirmed the expression of EGFP and mRFP in neural progenitors in the VZ and SVZ and in migrating cortical neurons (Fig. 1d–g). At this time point, most labeled cells in the VZ and SVZ co-expressed EGFP and mRFP (EGFP^+^mRFP^+^, Fig. 1h–k). Thus, majority of these cells might be labeled by transient reporter expressions. On the contrary, a certain number of progenitors in the VZ were selectively labeled with EGFP (EGFP^only^), suggesting that these cells were permanently labeled by successful genomic integration of the reporter vector. A few cells were mRFP single-positive (mRFP^only^), probably due to the depletion of the EGFP reporter vector following cell divisions. To trace sibling cell types derived from these labeled neural progenitors, we examined electroporated brains at postnatal day 34 (P34), allowing sufficient time for the generation of neuronal and glial cells from embryonic neural progenitors. We found a variety of labeled cells in the cortical and subcortical regions (n = 6 animals); in four samples (130910-1, -2, -3, and -4), all labeled cells were exclusively distributed in the neocortex (NCx), suggesting accurate electroporation of neural progenitors in the dorsal telencephalon. On the contrary, in two samples (140606 and 140821), scattered distribution of labeled cells was detected in the neocortex and striatum (NCx + Str). Thus, we speculated that neural progenitors in both dorsal and ventral telencephalon were electroporated in these samples. Labeled cells were classified into several cell types including neocortical pyramidal neurons and striatal stellate neurons, astrocytes, and ependymal cells, according to cellular morphology and the expression of cell type-specific markers (Fig. 2a–l and Supplementary Fig. S1, labeled neurons in 140606 and 140821 might have included interneurons derived from the subpallium.) Notably, the combination of fluorescent proteins was different among cell types: all combinations of fluorescent reporters (EGFP^only^, mRFP^only^, and EGFP^+^mRFP^+^) were detected in neurons in the neocortex or striatum (Figs 2a–c and 3), while astrocytes included exclusively EGFP^only^ cells, consistent with the transition from neurogenesis to gliogenesis during the embryonic and postnatal periods (Figs 2d–f and 3). As in the case of neurons, ependymal cells consisted of EGFP^only^, mRFP^only^ and EGFP^+^mRFP^+^ cells (Figs 2g–i and 3), suggesting that these cells were differentiated throughout embryonic to postnatal periods. The existence of mRFP^only^ cells might be due to inefficient integration or to silencing of the Tol2 vector. In 2 animals in which both pallial and subpallial neural progenitors were electroporated, EGFP and/or mRFP-positive neurons were localized in the granule cell layer of the olfactory bulb (Figs 2j,k and 3). In these samples, EGFP^only^ migrating neuroblasts were detected in the rostral migratory stream (RMS), which were supposed to be derived from postnatal neural stem cells (Figs 2j,l and 3). These data revealed that the progeny of neural progenitors in early developmental stages of the telencephalon also contributed to postnatal/adult SVZ neural stem cells, as previously reported^14,15^. Although adult neural stem cells were reported to exist on the dorsal side of the SVZ^22^, we could not detect labeled neuroblasts after electroporation into the pallial neural progenitors (Fig. 3 and data not shown). Thus, it is possible that early-stage neural progenitors in the dorsal telencephalon infrequently contribute to postnatal and adult neural stem cells. Likewise, oligodendrocyte-like cells were rarely labeled by either cortical or subcortical progenitor labeling (n = 1 cell in 6 samples, data not shown), in line with a recent report that spatiotemporally restricted progenitor pools contribute to oligodendrocyte generation^23^.

### Cumulative and non-cumulative labeling of neurons and non-neuronal cells in the developing mouse brain

Next, we compared the proportion of each cell type among total EGFP-positive progenies, which were labeled by the Tol2-reporter vector. In the P34 cerebrum (n = 6), the vast majority of EGFP-positive cells were neocortical or striatal neurons (90.4%, Supplementary Fig. S2). In contrast, the number of EGFP-positive astrocytes and ependymal cells was markedly smaller (astrocytes: 4.4%; ependymal cells: 2.8%, Supplementary Fig. S2). These data suggest that neural progenitors in early stages of the mouse telencephalon predominantly contribute to cortical and subcortical neurons.

**Figure 4:**
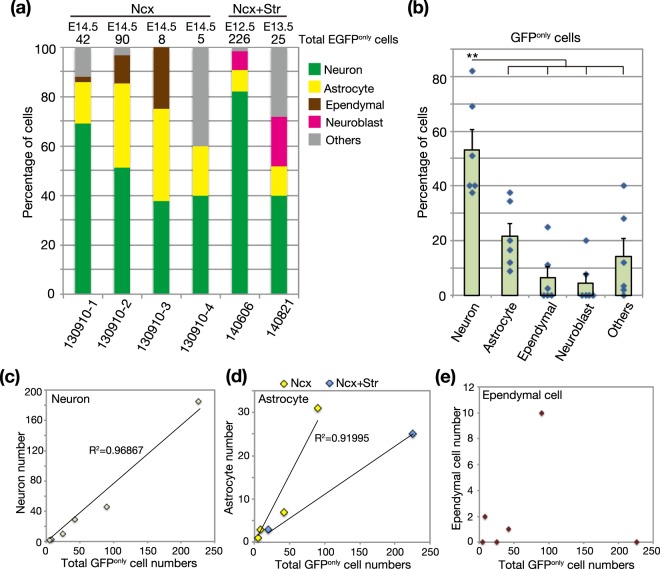
Cumulative and variable labeling of various cell types among EGFP^only^ cells in P34 mouse brains. (**a**,**b**) The proportion of various cell types among EGFP^only^ cells in P34 mouse cerebrum. In 4 samples, labeled cells were detected only in the neocortex (Ncx), while other 2 samples show a broad distribution of labeled cells in both the neocortex and striatum (Ncx and Str). The average ratio of neurons and astrocytes was 2.5:1 (**b**), in consistent with a previous report^21^. Data were expressed as mean ± SE. (**c**–**e**) Variations in numbers of neurons (**c**), astrocytes (**d**), and ependymal cells (**e**) according to numbers of GFP^only^ cells. Neurons and astrocytes were cumulatively labeled by the increases of labeling efficiency, while ependymal cell labeling was more fluctuated. **p < 0.01.

However, majority of EGFP-positive neurons was labeled by immediate expression of non-integrative reporter vector, which greatly outnumbered the proportion of neurons. Thus, we focused on EGFP^only^ cells that were presumably composed by permanently labeled cells. In EGFP^only^ cells, the proportion of neurons was still higher than that of astrocytes (neuron: 53.2 ± 7.46%; astrocyte: 21.5 ± 4.8%, Fig. 4b). We compared our data with a previous permanent tracing of neural progenitors by using *NestinCreER;Z/EG* mice^23^. The ratio of neurons and astrocytes in EGFP^only^ cells was highly consistent with a previous report (2.5:1), corroborating that numbers in EGFP^only^ cell types faithfully represents the lineage relationship of neurons and non-neuronal cells.

Total numbers of EGFP^only^ cells were variable, presumably due to the efficiency of electroporation and/or random genomic integration of the reporter vector (Fig. 4a,b). Nevertheless, we found cumulative labeling of neurons according to the total number of labeled progenies (R^2^ = 0.96867, Fig. 4c), regardless of embryonic stages or dorso-ventral positions of neural progenitors. This suggests that a large population of progenitors has similar potentials to generate neurons. On the contrary, numbers of astrocytes was more variable, although it showed a weak correlation with the total number of labeled cells (R^2^ = 0.358). However, quantification of two separate groups depending on the position of electroporation (NCx and NCx + Str) showed significant correlations of astrocyte numbers and labeling efficiency (Fig. 4d). Interestingly, these two groups revealed distinct linear functions, suggesting spatial differences in the gliogenic potentials of neural progenitors. Numbers of ependymal cells was highly fluctuated and did not show any positive correlation with labeling efficiency (Fig. 4e). These results also suggest limited number of neural progenitors generate ependymal cells, which resulted in stochastic labeling of this cell type in small number of population. As shown in Fig. 3, neuroblasts in the RMS were detected only in samples that contained progenies derived from subpallial progenitors (NE + Str; Fig. 4a), also implying spatial variability of neural progenitors that contribute to postnatal/adult neural stem cells.

### Sequential and cumulative labeling of neurons and glial cells in the developing chick brain

**Figure 5:**
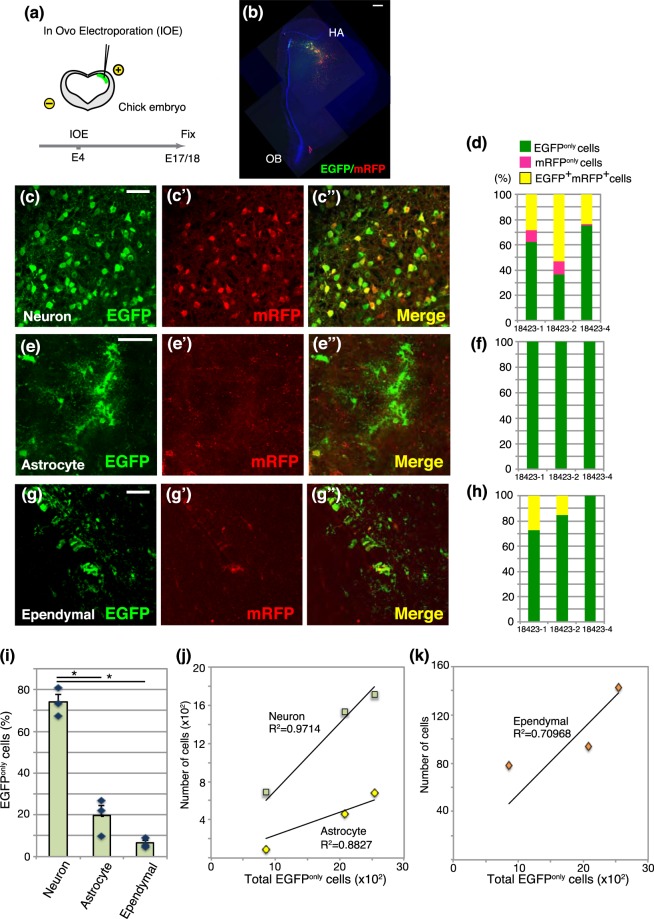
Population-level tracing of neural progenitors in the developing chick pallium. (**a**) *In ovo* electroporation of chick embryos. (**b**) Distributions of EGFP and/or mRFP-positive cells in E18 chick pallium. HA: Hyperpallium apicale, OB: olfactory bulb. (**c**–**g”**) EGFP and/or mRFP- labeled neurons (**c**–**c”**), astrocytes (**e**–**e”**), and ependymal cells (**g**–**g”**). (**d**,**f**,**h**) The proportion of EGFP and/or mRFP-positive cells in neurons (**d**), astrocytes (**f**), and ependymal cells (**h**) in individual samples. (**i**) The proportions of various cell types in GFP^only^ cells. Data were expressed as mean ± SE. (**j**,**k**) Variations in numbers of neurons (squares in **j**), astrocytes (diamonds in **j**), and ependymal cells (**k**) according to numbers of EGFP^only^ cells. *p < 0.05. Scale bars: 500 µm in b; 50 µm in (**c**,**e**,**g)**.

Avian brains harbor enormous numbers of neurons and glial cells, which underlie their sophisticated social behaviors and higher cognitive functions^24^. To investigate whether progenitor potentials for neurogenesis and gliogenesis are comparable in the developing mammalian and avian brains, we performed long-term tracing of chick embryonic neural progenitors by Tol2 transposon system. As in the case of the developing mouse neocortex, neurons and glial cells are sequentially generated in the developing chick pallium^25^. Thus, we electroporated reporter vectors into chick pallial neural progenitors at E4 (Hamburger and Hamilton stages HH23-24), the onset of pallial neurogenesis, and examined the brain at E17 or E18, at which glial cells are abundantly generated (Fig. 5a). We confirmed EGFP and/or mRFP-positive cells in the chick pallium, which includes the hyperpallium, mesopallium and nidopallium (Fig. 5b and data not shown). Fluorescent reporter-positive cells were classified into neurons and non-neuronal cells by their morphology and the expression of Satb2: the former were distinguished by a Satb2-positive larger soma with multiple processes^26^, while the majority of the latter population were ramified astrocytic cells that were frequently associated with blood vessels and Satb2 negative (Supplementary Fig. S3). We confirmed labeled neurons in the chick pallium consisted of EGFP^only^, mRFP^only^, and EGFP^+^mRFP^+^ cells (Fig. 5c-c”,d). By contrast, astrocytes were composed exclusively of EGFP^only^ cells, as in the case of tracing in the developing murine cerebrum (Fig. 5e-e”,f). Ependymal cells (or remaining radial glial cells) were also labeled in the pallial VZ with various combinations of fluorescent reporter proteins (Fig. 5g-g”,h). Quantitative analysis of these three cell types among EGFP^only^ cells revealed that the majority of labeled cells were pallial neurons (73.97 ± 3.86%), while small numbers of labeled cells were astrocytes or ependymal cells (19.59 ± 5.02%; 6.42 ± 1.39%, respectively, Fig. 5i,j). These data suggest that neural progenitors in early stages of the chick pallium predominantly contribute to neurogenesis than gliogenesis. Furthermore, neurons and astrocytes were cumulatively labeled according to total number of labeled cells (Fig. 5j), suggesting constant probabilities of neuron and astrocyte generations from avian neural progenitors, as in the case of mouse brains. On the contrary, the number of ependymal cells was less correlated with labeling efficiency (Fig. 5k), suggesting more fluctuated generation of this cell type from individual chick neural progenitors. We could not detect RMS-like streams of migrating neuroblasts, implicating that chick pallial progenitors rarely contribute to persistent neurogenesis (data not shown). Thus, differential potentials of neural progenitors are evolutionarily conserved among the developing amniote brain.

## Discussion

Transposon-mediated genome integrative vectors are valuable for performing permanent cell tracking in a variety of organisms^12,13,17,19–21,26^. Combinatorial use of non-integrative vectors can be used to distinguish several cell types depending on their differentiation timings. Indeed, in the present study, we observed differential combinations of fluorescent reporters in neurons and non-neuronal cells; in particular, exclusive composition of astrocytes by EGFP^only^ cells revealed that gliogenic progenitors were persistently labeled after the end of neurogenesis, and thus could not be traced by electroporation of non-integrative vectors. In this context, it is noteworthy that ependymal cells, like cortical and subcortical neurons, consisted of multiple combinations of fluorescent colors. Thus, subpopulations of ependymal cells are committed at early stages of embryogenesis, as previously suggested^27^, despite the postnatal appearance of this cell type on the surface of the lateral ventricle.

Importantly, our population-level tracing suggested that a large population of neural progenitors in the early embryonic stage has similar probabilities to generate cortical or subcortical neurons. Higher number of neurons than astrocytes in the labeled cells implied that majority of progenitors in the E12-14 telencephalon is neurogenic progenitors. A recent genetic fate mapping of mouse cortical progenitors by mosaic analysis of double markers (MADM) revealed that approximately 16% of cortical radial glial progenitors proceed to gliogenesis at the end of neurogenesis^9^. Our results support the hypothesis that coherent and deterministic programs operate neurogenesis and gliogenesis in individual progenitors. On the contrary, we suggest a limited population of progenitors generates ependymal cells. A previous study indicated that final mitosis of ependymal cell progenitors occurred at E13-14^27^, suggesting that a small subset of progenitors in early embryonic stages are already committed to generate ependymal cells. Alternatively, progenitors that generate ependymal cells could be spatially biased in the telencephalon. Recent studies have shown that distinct progenitor domains in the mouse embryonic brain contributed to non-uniform distributions of neurons and astrocytes^12,13^. Altogether, it is possible that the heterogeneity of progenitor populations is dynamically changed through neurogenic and gliogenic periods in a spatio-temporally controlled manner.

Postnatal neural stem cells are broadly distributed in the SVZ of the lateral ventricle that derived from both embryonic pallium and subpallium^15,22^. However, we could not find labeled neuroblasts in RMS by targeting pallial neural progenitors, suggesting that the number of neural progenitors that contribute to postnatal neurogenesis might be extremely lower in the embryonic pallium compared to those in the subpallium. Consistently, recent studies have reported that slowly dividing embryonic neural progenitors enriched at the lateral ganglionic eminence, a part of the subpallium, are the origin of postnatal and adult neural stem cells^14^.

The glia/neuron ratio in the neocortex is highly variable among species and is inversely correlated with neuronal density^28^. Notably, neuronal densities in avian pallium are much higher than those in mammalian neocortex with the same mass^24^. We demonstrated dominant neurogenic potential of neural progenitors in the developing chick brain, although the timings of analysis were not comparable between mice and chick. Notably, a previous lineage tracing in the P14 and P21 chick telencephalon by using a library of retroviral vectors^29^ indicated higher proportion of astrocytes in total clonal siblings (42.8%) than our analysis. Thus, it is possible that we underestimate the gliogenic potential in the developing chick brains, although a lower recovery rate of genomic amplification in previous clonal analyses^30^ (less than 10% of clones was successfully amplified and sequenced) resulted in substantially biased estimations in the proportion of cell types.

Because parenchymal astrocytes have not been identified in the mature amphibian and reptilian brains^4,31^, various neuronal and glial subtypes are thought to have evolved independently in mammalian and avian lineages. Our results suggest that basic gliogenic programs already existed in common ancestors of amniotes; alternatively, distinct glial cell types in mammals and birds may have independently appeared from ependymoglial cells that shared characteristics with astrocytes and neural stem cells^32^.

## Methods

### Animals

Pregnant female mice (ICR background, 8 weeks) were purchased from Japan SLC. Inc. The noon on day of the vaginal plague was designated E0.5. Fertilized chicken eggs were purchased from a local farm (Yamagishi) and incubated at 37 °C. All experimental procedures were approved by the Animal Experiment Committee at Kyoto Prefectural University of Medicine (M29-102; M29-111) and performed according to the guidelines.

### *In utero* and *in ovo* electroporation

*In utero* and *in ovo* electroporation were performed according to previous reports^26,33^. For *in utero* electroporation, pregnant mice (E12-14) were anesthetized with isofluran (Escain, Mylan Pharmacy) using an anesthesia unit (Univentor 410, BRC; gas flow was 250–300 mL/h, 3.0%). Approximately 0.1–0.5 µL of DNA solution containing pCAGGS-T2TP, pT2AL-CAGGS-EGFP, and pCAGGS-mRFP (0.1 µg/µL of each plasmid) was injected into the lateral ventricle of embryonic mouse or chicken brains, and square electric pulses were applied by an electroporator (CUY21 Edit II, BEX, Japan) through forceps-type electrodes (CUY650P3 for mouse embryos and CUY200S for chick embryos).

### Tissue processing

Embryonic mouse and chick brains were fixed by immersion in 4% paraformasldehyde [PFA; dissolved in phosphate buffered saline (PBS)]. Postnatal mouse brains were fixed by transcardial perfusion with 4% PFA under deep anesthesia with pentobarbital. After washing with PBS, brains were sectioned at 50 µm thickness in a vibrating microtome (VT1000S, Leica) or cryoprotected with 20% sucrose for sectioning at 20 µm thickness in a cryostat (CM1850, Leica).

### Immunohistochemstry

Immunohistochemistry was performed according to previous reports by using following primary antibodies: anti-GFP (rat monoclonal, Nacarai Tesque, 1:1000; rabbit polyclonal, Millipore, 1:1000), anti-NeuN (rabbit polyclonal, Millipore, 1:1000), anti-S100 (rabbit polyclonal, abcam, 1:1000), anti-GFAP (mouse monoclonal, Sigma-Aldrich, 1:1000), anti-Satb2 (mouse monoclonal, abcam, 1:1000), and anti-Dcx (goat polyclonal, Santa Cruz, 1:1000) antibodies. After washing with tris-buffered saline with Tween-20 (TBST), the sections were incubated with secondary antibodies conjugated with Alexa-Fluor 488, 594 or 633 (Thermo Fisher Scientific, 1:500), and Hoechest 33258 (Thermo Fisher Scientific, 1:1000) for nuclear staining. Coverslipped samples were examined with a fluorescent microscope (BX51, Olympus) or a laser confocal microscope (FV1000D, Olympus).

### Imaging and data analysis

The number of EGFP- and/or mRFP-positive cells in postnatal mouse brains was quantified by examining all brain sections containing labeled cells under a fluorescence microscope. The number of EGFP- and/or mRFP-positive cells in chick brains was counted by capturing confocal images with a 40X objective, and at least three sections from each sample were examined. Captured images were processed with ImageJ (NIH) and Adobe Photoshop CC. The number of EGFP- and/or mRFP-positive cells in each sample is represented in Figs 3,4 and 5. Correlations between the number of each cell type and total number of labeled progenies were calculated by Microsoft Excel 2011. Statistical significance was calculated by Student’s *t*-test and Turkey’s multiple test.

## Supplementary information

Supplementary Information
