# Supplementary material for: Differential potentials of neural progenitors for the generation of neurons and non-neuronal cells in the developing amniote brain
Source: Sci Rep. 2019 Mar 14;9:4514. doi: 10.1038/s41598-019-40599-2 (PMC6418204; doi:10.1038/s41598-019-40599-2)
Supplement: Supplementary file 1 — Supplementary Information [file 41598_2019_40599_MOESM1_ESM.docx]

**Supplemental Information**

**Differential potentials of neural progenitors for the generation of neurons and non-neuronal cells in the developing amniote brain**

Yuki Hashimoto^1^, Hitoshi Gotoh^1^, Katsuhiko Ono^1^, Tadashi Nomura^1^*

^1^Developmental Neurobiology, Graduate School of Medicine, Kyoto Prefectural University of Medicine, 1-5 Shimogamo-hangi cho, Sakyoku, Kyoto 606-0823, Japan

*Correspondence: [tadnom@koto.kpu-m.ac.jp](mailto:tadnom@koto.kpu-m.ac.jp)

**Supplementary Figure S1. The expression of cell type-specific markers in EGFP-positive cells in P34 mouse brains.**

Immunohistochemistry with anti-NeuN (A), anti-GFAP (B), and anti-S100 (C, D) antibodies reveal a cortical neuron (A), fibrous astrocyte (B), protoplasmic astrocyte (C), and ependymal cell (D). Scale bars: 25 µm.

**Supplementary Figure S2. Cell type compositions in EGFP-positive cells (including EGFP^+^/mRFP^+^ and EGFP^only^ cells) in P34 mouse cerebrum.**

(A, B) The proportion of neurons, astrocytes, ependymal cells, neuroblasts, and others (unclassified cells) among EGFP-positive cells in individual samples (A) and mean ± SE (B, *p<0.05). (C, D, E) Variations in numbers of neurons (C), astorocytes (D), and ependymal cells (E) according to total numbers of labeled cells. P<0.001(C).

**Supplementary Figure S3. The expression of Satb2 in EGFP-positive cells in E18 chick pallium.**

Immunohistochemistry with anti-Satb2 antibody indicates pallial projection neurons (A-D). In contrast, EGFP-positive astrocytic cells were not labeled by Satb2. Scale bar: 50 µm.
